# Cutaneous Lesions of Multiple Myeloma of the Lower Extremity Masquerading as Squamous Cell Carcinoma

**DOI:** 10.7759/cureus.11313

**Published:** 2020-11-03

**Authors:** Courtney A Clutter, Savina Aneja, Doina Ivan, Ana Ciurea, Sirunya Silapunt

**Affiliations:** 1 Internal Medicine, San Antonio Military Medical Center, San Antonio, USA; 2 Advanced Dermatology and Cosmetic Surgery, University of Central Florida, Orlando, USA; 3 Pathology, University of Texas MD Anderson Cancer Center, Houston, USA; 4 Dermatology, University of Texas MD Anderson Cancer Center, Houston, USA; 5 Dermatology, University of Texas McGovern Medical School at Houston, Houston, USA

**Keywords:** cutaneous metastases, cutaneous myeloma, multiple myeloma, squamous cell carcinoma

## Abstract

Cutaneous involvement in multiple myeloma (MM) is a rare manifestation, being more prevalent in patients with aggressive subtypes, and refractory to standard therapies. Due to the rarity of this diagnosis, the reported clinical characteristics have been protean and relatively non-specific. Lower extremity involvement of cutaneous MM is an uncommon anatomical location for this diagnosis. We present a patient with refractory IgG lambda MM, and a past medical history of squamous cell carcinoma of the lower extremities who developed cutaneous MM in his lower leg. At the time of initial evaluation, the lesions mimicked squamous cell carcinoma, posing a diagnostic challenge. Histopathological and immunohistochemical studies confirmed cutaneous involvement by multiple myeloma. There needs to be a high clinical suspicion for cutaneous MM in patients with MM presenting with new skin lesions.

## Introduction

Multiple myeloma (MM) can uncommonly manifest as cutaneous lesions. The prevalence of cutaneous involvement is estimated to be less than 2% of patients with systemic MM [1]. Cutaneous involvement in MM often presents at advanced stages of the disease due to increased tumor cell burden [2]. Cutaneous lesions could be the result of direct extension from underlying bone lesions or cutaneous metastases in the absence of underlying osseous lesions [2]. There is a wide anatomical distribution of these lesions, with the most common locations being the trunk and abdomen [1,2]. Limited medical literature has discussed cutaneous involvement in MM. We report a rare presentation of cutaneous MM mimicking squamous cell carcinoma of the lower leg, in a patient with a past medical history of squamous cell carcinoma of the lower extremities.

## Case presentation

A 63-year-old male with MM and a past medical history of psoriasis, squamous cell carcinoma of the left shin treated with Mohs surgery, cellulitis, coronary artery disease, diabetes and arthritis was seen by the inpatient dermatology service for the lesion on his right lower leg. Two years prior to admission, he had been diagnosed with IgG lambda MM with an initial bone marrow biopsy showing plasma cells compromising 80% of the marrow. His bone scan showed involvement of right and left humerus. The patient was initially treated with a combination of lenalidomide, bortezomib, dexamethasone and panobinostat. Due to refractory disease, he was later placed on treatment with modified CVAD (cyclophosphamide, vincristine, doxorubicin also known by its trade name: Adriamycin and dexamethasone) with vincristine omitted and substituted with bortezomib followed by an autologous stem cell transplant. In the setting of relapsed disease, he was treated with carfilzomib and panobinostat, and later Hyper-CVAD and dexamethasone.

**Figure 1:**
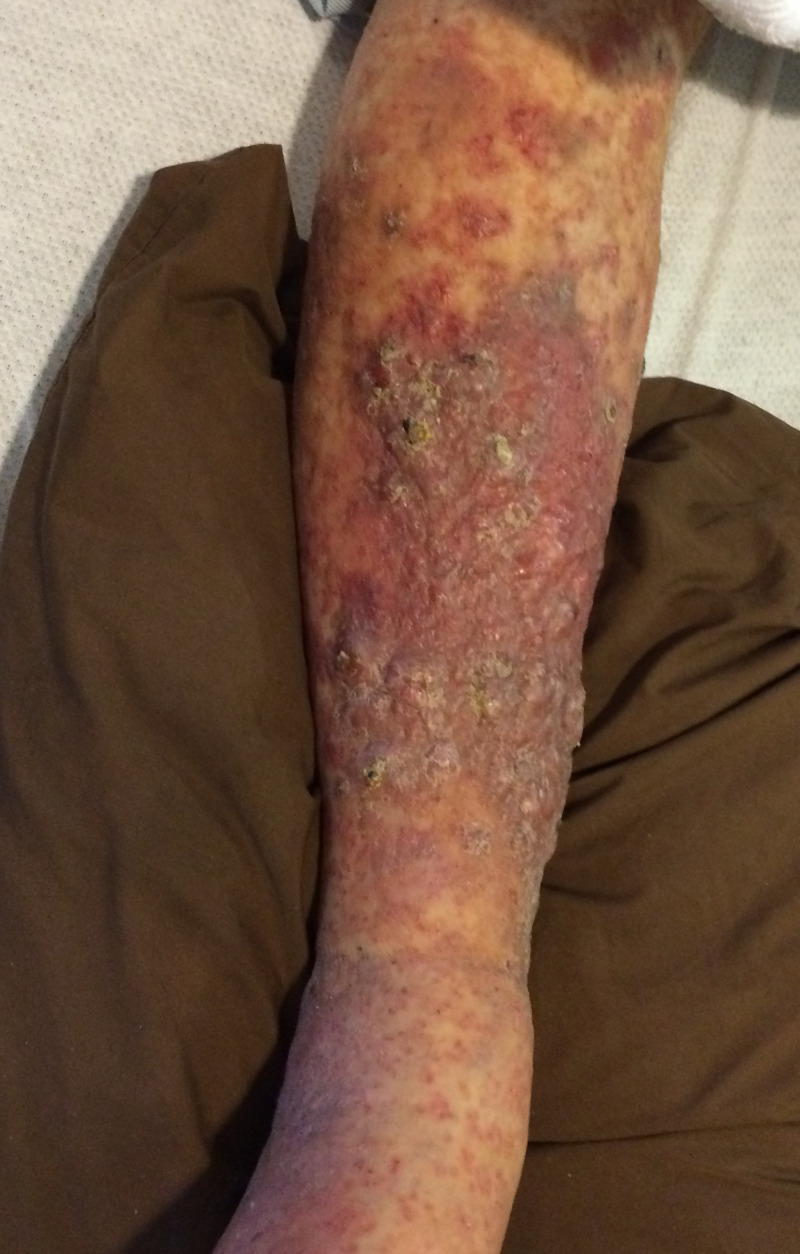
Hyperkeratotic verrucous erythematous plaques on the right lower leg.

**Figure 2:**
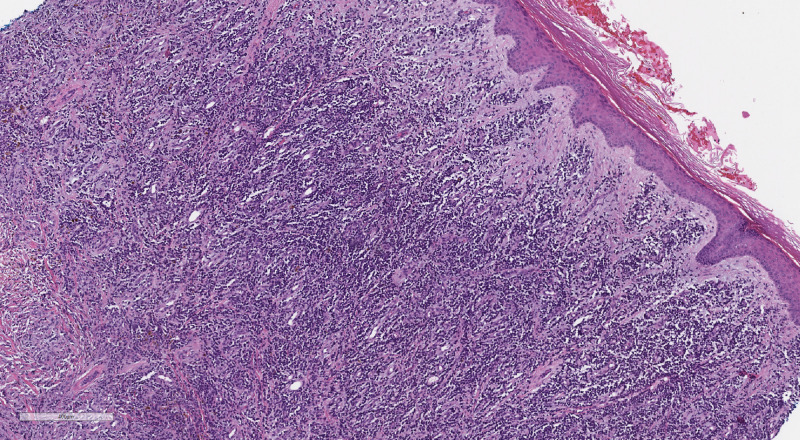
Extensive dermal infiltrate of plasma cells with cytologic atypia, binucleation and scattered mitotic figures (Hematoxylin-eosin stain, original magnificationx10).

**Figure 3:**
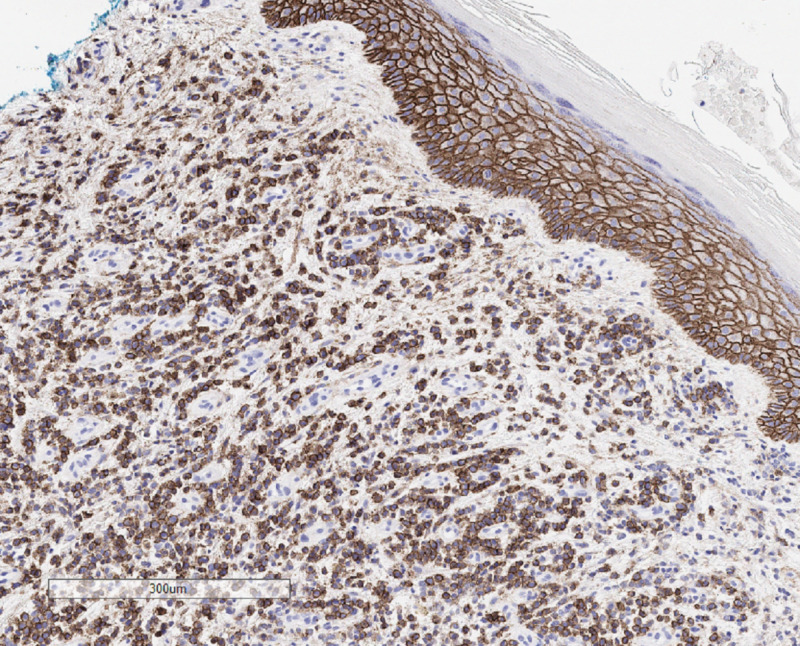
Dermal cells label diffusely with CD138.

**Figure 4:**
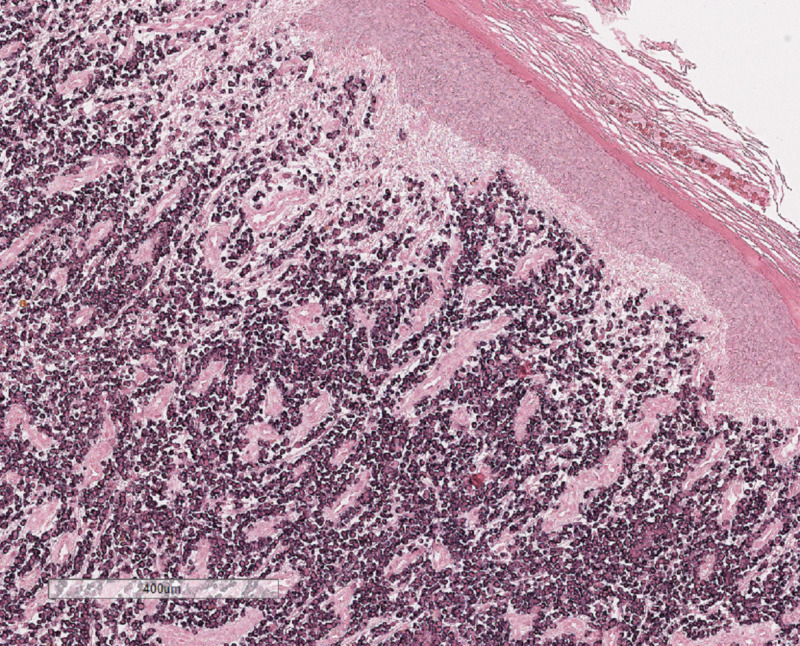
The majority of the cells are positive for lambda light chain.

Several weeks after receiving chemotherapy, the patient reported enlarging skin lesions and pain on the right lower leg. On physical exam he was noted to have verrucous hyperkeratotic erythematous plaques on the right shin (Figure 1). The differential diagnosis included squamous cell carcinoma, psoriasis, deep fungal infection, cutaneous amyloidosis, chronic eczema, and metastatic disease. The patient had psoriasis on upper and lower extremities which was well-controlled with topical steroids. His psoriasis had not recurred since he started chemotherapy for multiple myeloma. Two punch biopsies were performed to further characterize the lesion. Specimens were sent to pathology for H&E and to microbiology for culture. Microbiology cultures for fungal or bacterial organisms, and acid-fast bacilli were negative. Histological examination revealed an extensive dermal plasma cell infiltrate composed of cells with cytologic atypia, binucleation and scattered mitotic figures (Figure 2). Immunohistochemical studies demonstrated that the dermal cells were labeled diffusely with CD138 and there was marked light chain restriction with the majority of the cells positive for lambda and rare cells positive for kappa (Figures 3 and 4). These findings were consistent with cutaneous involvement by MM.

The patient was treated with daratumumab, dexamethasone and pomalidomide. Despite treatment, the patient died 1 month after the diagnosis of cutaneous involvement, due to progression of the systemic disease and treatment-related pancytopenia.

## Discussion

Due to the rarity of cutaneous involvement in MM, the reported clinical characteristics have been protean and relatively non-specific. In the limited medical literature regarding this subject, the presentation of cutaneous involvement in MM typically includes erythematous and violaceous papules, nodules, or plaques in varying sizes [1,2]. 

Cutaneous lesions in MM can involve any part of the skin, but favor the trunk and abdomen [1,2]. Involvement on the extremities, as in our patient, is unusual [2].

Given the patient’s history of squamous cell carcinoma of the lower extremity and his immunosuppressive therapy, the lesions on his lower leg were postulated to be of squamous cell carcinoma by the primary service. The verrucous plaques with hyperkeratosis and erythema mimicked the appearance of squamous cell carcinoma. However, the aggressive nature of the patient’s MM and plaque-like distribution led to the consideration of cutaneous involvement by MM.

There should be a low threshold to biopsy patients with refractory MM who develop new skin lesions as histopathological and immunohistochemical studies are essential in confirming the diagnosis of a clonal plasma cell process, and distinguishing this from other cutaneous malignancies such as squamous cell carcinoma, B-cell lymphoma, or T-cell lymphoma. Histopathological studies will demonstrate a nodular or diffuse interstitial infiltrate of atypical plasma cells [1,3]. Immunohistochemical studies show CD138, CD79a, and epithelial membrane antigen expression in the neoplastic cells [2,4]. All major immunoglobulin classes, except for IgE, have been reported in cutaneous MM [4,5] with IgG being the most common subtype associated with cutaneous lesions [2,6-7].

The patient’s bone scan showed involvement of right and left humerus. His skin lesions were likely due to cutaneous metastases rather than direct extension from underlying bone lesion.

Cutaneous involvement of MM is more prevalent in patients with more aggressive subtypes, refractory to standard therapies, and is indicative of a poor prognosis [1,3]. The majority of patients with cutaneous involvement die within 1 year after the appearance of cutaneous lesions [1,2,6], thus making this diagnosis important for prognostic purposes. The aggressive nature of our patient’s disease was evident after failing multiple chemotherapy regimens and autologous stem cell transplant. Shortly after developing cutaneous MM, our patient passed away, highlighting the poor prognosis that cutaneous involvement of MM portends.

## Conclusions

Multiple myeloma rarely presents with cutaneous involvement and therefore, there is a paucity of medical literature regarding the subject matter. In order to gain a more robust understanding of cutaneous involvement in MM, clinicians must continue to share unique patient presentations of this entity. Having a high clinical suspicion of new verrucous papules, plaques and nodules presenting in patients with MM, especially those with aggressive or advanced disease is paramount to making this diagnosis, which has important prognostic implications. Histopathological and immunohistochemical studies are essential to confirming the diagnosis, and differentiating it from other malignancies such as squamous cell carcinoma, which was in the clinical differential diagnosis.
